# The pollination syndrome of parasitic plants depends on the environment

**DOI:** 10.1093/pcp/pcag023

**Published:** 2026-02-17

**Authors:** Peter Tóth, Sjors Huizinga, Harro Bouwmeester

**Affiliations:** bioTomal, Rúbaň, 176, 94136 Rúbaň, Slovakia; Swammerdam Institute for Life Sciences, University of Amsterdam, 1090 GE Amsterdam, the Netherlands; Swammerdam Institute for Life Sciences, University of Amsterdam, 1090 GE Amsterdam, the Netherlands

**Keywords:** floral volatiles, GC–MS, Orobanchaceae, *Orobanche flava*, pollinator-mediated selection, volatile organic compounds

## Abstract

Plants interact ubiquitously with organisms surrounding them through chemical communication, for example through the emission of volatile organic compounds (VOCs) by flowers, to attract pollinators. To attain specificity in this relationship, plants may evolve different VOCs for different pollinators. In the present study we investigated the flower VOCs of the root parasitic butterbur broomrape (*Orobanche flava*), which parasitizes several different *Petasites* spp. at geographically different locations in Slovakia, and analyzed the corresponding community of pollinators, some of which have never been reported before for *O. flava*. There were intriguing differences in floral scent phenotype as well as pollinator community composition between the locations, suggesting the existence of different ecotypes of *O. flava*. We discuss this variation in floral VOCs and compare the results with other studies investigating intraspecific variability in floral scent. The differences in floral scent chemistry and corresponding pollinator species between broomrapes in different regions suggest pollinator-mediated selection across the geographical distribution of this parasitic plant species.

## Introduction

Parasitic plants are plants that parasitize on other plants. As a result, they have partially or completely lost photosynthesis, and in other aspects evolved a phenotype and physiology adapted to their parasitic lifestyle (Yoshida et al. 2016). Of all known plant species, only 1% are parasitic, but still, this represents 292 genera and about 4750 species (Nickrent and Musselman 2016). Parasitism by these parasitic plants reduces the productivity and reproduction of the host (Press and Phoenix 2005), into which the parasite has penetrated using a haustorium (Heide-Jørgensen 2008, Yoshida et al. 2016, Zhang et al. 2016, Xiang et al. 2018, Nickrent 2020) and can even result in its death (Aukema 2003, Joel 2013). Nevertheless, parasitic plants play a significant role in ecosystems, positively affecting species diversity, limiting dominant species, altering nutrient cycling and biomass levels (Press and Phoenix 2005, Schneeweiss 2007, Graffis and Kneitel 2015), and even influencing the ecosystem’s fauna (Bennetts et al. 1996, Watson 2001). They may thus be considered ecosystem engineers (Jones et al. 1994). On the other hand, they are a problem in agriculture (Qasem 2006, Kebede and Ayana 2018, Fernández-Aparicio et al. 2020), although only ~25 species are considered harmful (Nickrent et al. 2016). Moreover, most of the damage is caused by representatives from just five genera: *Cuscuta*, *Arceuthobium*, *Striga, Orobanche*, and *Phelipanche* (Nickrent 2002). The damage caused by broomrapes (*Orobanche* and *Phelipanche* spp.) ranges from 5% to 100% (Abang et al. 2007). The Orobanchaceae, to which *Striga* and the broomrapes belong, is the largest parasitic family with 102 genera and more than 2100 species (Heide-Jørgensen 2008, Nickrent 2020). They parasitize on the roots of other vascular plants (Piwowarczyk et al. 2018). The seeds of these parasites only germinate when exposed to host root–exuded “germination stimulants” (such as strigolactones), ensuring this occurs only in the vicinity of a host root (Yoneyama et al. 2010, 2013, Bouwmeester et al. 2021) as they would die if they do not attach within a couple of days (Vurro et al. 2019). Once attached to a host root, they grow at the expense of the host, emerge above ground, flower, and set seed (Pérez-De-Luque et al. 2010).

Broomrapes are pollinated by insects, mostly by bumblebees and bees (Apidae), but also by species from the Vespidae, Halictidae, Colletidae, and Syrphidae (Tóth et al. 2013, Piwowarczyk et al. 2015, Konarska and Chmielewski 2020). *Orobanche* is one of the taxonomically most problematic genera due to the high degree of phenotypic variability (Konarska and Chmielewski 2020). Seed micromorphology (Plaza et al. 2004), pollen (Piwowarczyk et al. 2014, Zare et al. 2014), petals (Piwowarczyk and Kasińska 2017), and flower volatile organic compounds (VOCs) (Tóth et al. 2016) can, however, be used to aid species identification. One of these problematic broomrapes in this regard is butterbur broomrape (*Orobanche flava*). Its hosts are butterburs (*Petasites* spp.) of the family Asteraceae: *Petasites albus*, *Petasites kablikianus*, *Petasites hybridus*, and *Petasites paradoxus*. Butterbur broomrape can also attack other genera in the Asteraceae: *Adenostyles*, *Senecio*, and *Tussilago* spp. (Nikolov 2020) and thus is considered oligophagous (Piwowarczyk et al. 2018). *Orobanche flava* is a central European mountain species, mainly from the Alpine-Carpathian region. Until recently it was also considered an African species, since *O. flava* var. *doriae* grows in the Atlas Mountains. However, this plant is a parasite of *Senecio doria* and has now been classified as a separate species, *Orobanche doriae* (Emb. & Maire) Ó. Sánchez & Piwow., which is endemic to Africa (Piwowarczyk 2014, Piwowarczyk et al. 2023). Butterbur broomrape grows on floodplains along mountain streams and roads in limestone, dolomite, or flysch areas of the Carpathians or on the edge of forests. It prefers moist to wet, nutrient-rich, humus-rich, alkaline to neutral soils (Zázvorka 1997). Its distribution is relatively homogeneous in its habitats. Variability in flower color and in the phenotype of the whole plant is common, even within one population, making identification troublesome. The most common flower colors are ochre, yellowish, and light brown, but light yellow, pink, raspberry, or light red also occur (Piwowarczyk 2014).

In earlier work we showed that within the broomrapes there is considerable variability in the VOCs emitted by the flowers (Tóth et al. 2016). Plants synthesize and secrete a multitude of VOCs that play a key role in the interaction (communication) of plants with their environment (Bouwmeester et al. 2019). The amounts and relative proportions of VOCs in an emitted odor represent extraordinarily complex signals (Holopainen and Blande 2012, R. Karban et al. 2014a). VOCs are lipophilic compounds with low molecular weight. More than 1700 VOCs have been identified in the 90 angiosperm families (Picazo-Aragonés et al. 2020). Some VOCs are common to all plants; others are characteristic of one or a few related taxa (Pichersky and Gershenzon 2002). VOCs play a role in the attraction of pollinators, serve to defend plants and/or function as repellents to pathogens and pests, reduce the number of herbivorous insects by attracting parasitoids and predators, and contribute to signaling between plants (Heil 2014, Bouwmeester et al. 2019). The pollination syndrome is the key role of floral VOCs. The main reason is to provide the best possible “advertisement” for pollinators. More than 85% of all flowering plant species benefit from pollination (Ollerton et al. 2011). Most pollinators are attracted to the floral rewards offered by plants (nectar, pollen, or oils), with VOCs providing information on the location, quantity, and quality of these floral rewards. Plants must therefore attract and compete for the attention of pollinators to receive their services (pollination) (Howell and Alarcón 2007, Farré-Armengol et al. 2013). The composition of floral volatiles, as well as their total production, changes throughout the life span of the flower, depending on age, pollination status, environmental conditions, and daily endogenous rhythms. Flowers usually emit maximum amounts of VOCs when they are ready for pollination and when potential pollinators are active (Negre et al. 2003, Dötterl et al. 2012, Friberg et al. 2014, Jürgens et al. 2014, Junker 2016). Some plant species use deception or food mimicry (Schiestl et al. 2003). For instance, certain orchid species (Orchidaceae) use alarm pheromones to attract hoverflies for pollination (Stökl et al. 2011). Yet other plants may secrete flower VOCs reminiscent of carrion or dung odors (Niet et al. 2011). The parasitic plant *Cynomorium songaricum*, for example, releases volatiles such as *p*-cresol, indole, dimethyldisulfide, and 1-octen-3-ol to mimic rotting meat and attract dipterans (Wang et al. 2021). Finally, flower VOCs may also be repellent. Temperate flowers, for example, have been shown to repel many common European ants or deter unwanted florivores (Willmer et al. 2009, Huang et al. 2012, Muhlemann et al. 2014, Junker 2016). In the broomrapes, floral VOCs are also abundant (Tóth et al. 2016). They are highly species-specific and can even be used to recognize individual species. Intriguingly, the loss of certain floral VOCs in weedy broomrapes seems to have accompanied the loss of fertilization by social wasps and bumblebees (Tóth et al. 2016). However, nothing is known about the role of floral VOCs in pollinator attraction in broomrapes in natural ecosystems. In the present study, we therefore set out to analyze the floral VOCs of butterbur broomrape as well as the pollinators visiting them, across several different ecosystems. Our objective was to determine whether there is a relationship between variation in flower VOC composition and the attraction of different pollinator communities.

## Results

Comparison of the mass spectra with mass spectral libraries allowed the tentative identification of 135 volatile organic compounds (Supplementary Table S1). All the *O. flava* ecotypes produced these VOCs, but in different ratios. The most important VOCs out of the 135 appeared to be toluene, methyl 3-methylbutanoate (methyl isovalerate), nonanal, and tetracosane, which were described by Tóth et al. (2016) as general broomrape VOCs occurring in the floral VOCs of all broomrapes analyzed.

**Figure 1:**
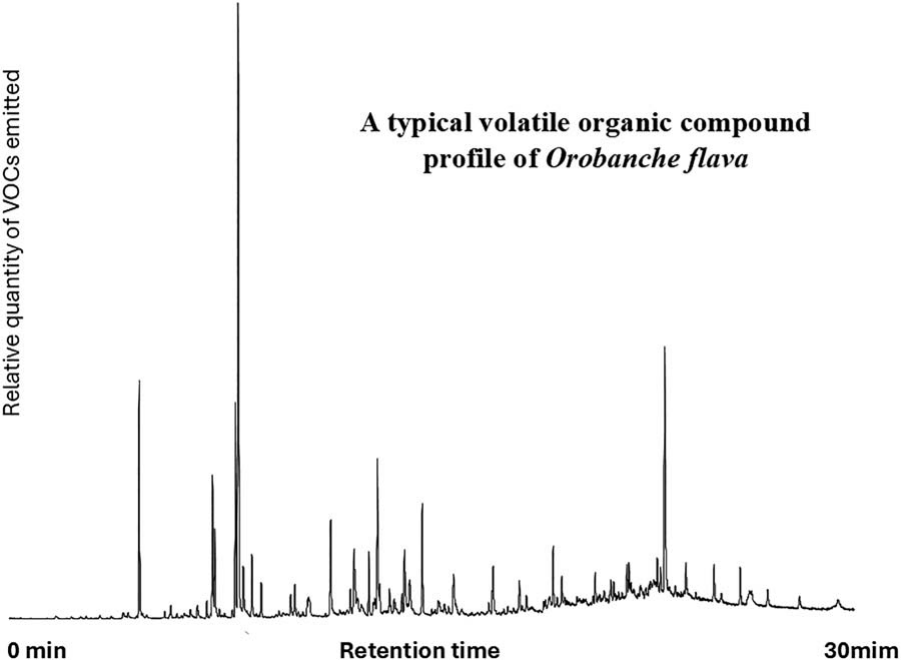
Typical VOC profile of *O. flava* at all the sites; changes are in VOC ratios.

The spectrum of *O. flava* VOCs was very diverse, including representatives of at least 12 functional groups: alcohols, aldehydes, amines, hydrocarbons (including monoterpenoids and sesquiterpenoids), aromatic hydrocarbons, carboxylic acids, esters, furans, ketones, phenols, sulfur compounds, and various other functional groups. The most dominant were ethyl acetate, isopentyl alcohol, toluene, methyl isovalerate, ethyl benzene, styrene, nonanal, and isopropyl myristate (Fig. 1).

Many of the compounds that we detected are known as semiochemicals with behavioral functions (e.g. kairomones, attractants, and pheromones). Pentane-2,4-dione and 2-decen-1-ol are the notable exceptions among the VOCs recorded, as they are not known from any other plants, including broomrapes (El-Sayed 2024).

**Figure 2:**
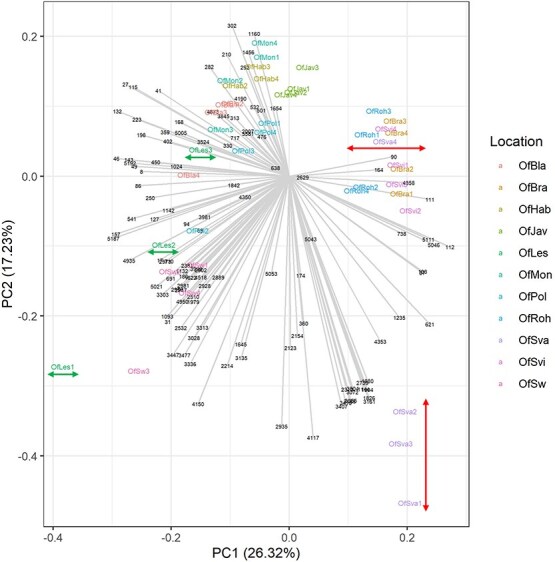
PCA of the floral VOCs of *O. flava* growing in 11 separate locations. PCA was done with compounds showing a significant difference between the locations (*P* < 0.05 from ANOVA test) using GeneMath XT. Letters represent single locations. OfSvi, Svidovo Valley; OfSva, Svarín Valley; OfBra, Bránica Valley; OfRoh, Roháčska Valley OfLes, Lesná Valley; OfSw, Slaná voda; OfBla, Blatnická Valley; OfPol, Polhoranka Valley; OfHab, Blatná Valley; OfMon, Monková Valley; OfJav, Javorová Valley.

**Figure 3:**
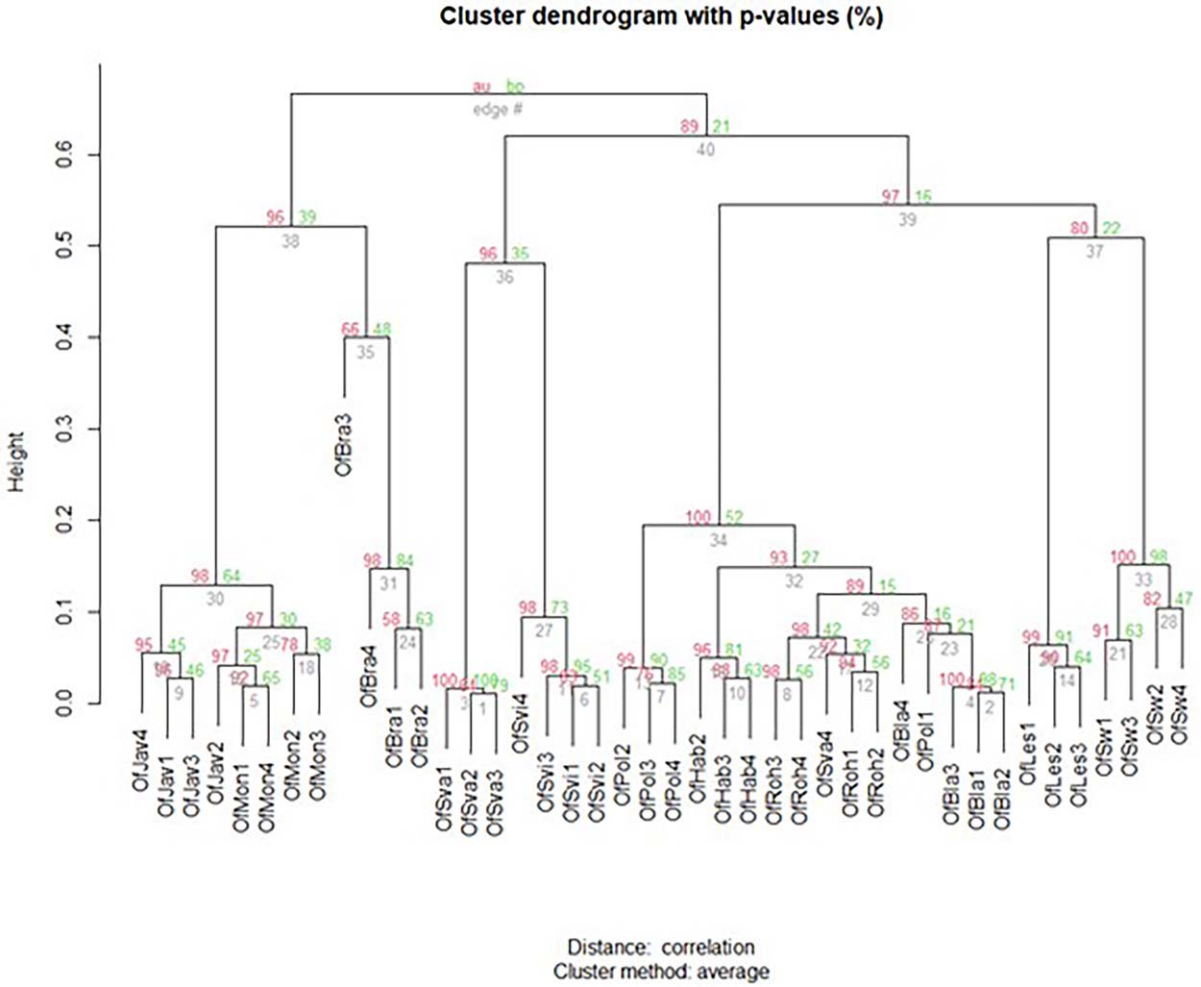
HCD of the flower VOC profiles of *O. flava* plants growing in 11 separate locations. HCD was performed with R and based on PCA of significantly different flower blends. Numbers at nodes are bootstrap probability *P*-value (BP, right green numbers) and approximately unbiased *P*-values (AU, left red numbers).

Principal component analysis (PCA) of the VOC data collected at the 11 separate locations showed that replicate samples were clustered together, demonstrating the reproducibility of the method (Figs. 2 and 3). Although they all belong to the same species, the samples were separated according to their location. This shows that the composition of the floral VOC blend of butterbur broomrape differs at these separate locations. Intriguingly, some samples clustered closely together even though they are from sites that are far apart and from different mountain ranges (Table 1). Completely separated ecotypes seem to be Svarín Valley (OfSva), Slaná voda (OfSw), and Lesná Valley (OfLes). The PCA plot also shows the VOCs that are driving the separate clustering of *O. flava* ecotypes (Fig. 2). Sample OfSva4 clusters together with OfBra, OfRoh, and OfSvi, perhaps because of their very similar big-valley habitats. Sample OfLes3 is quite far from the two other samples because of its weak signal (Fig. 2).

**Table 1:** Ecotypes of *O. flava*, occurrence in mountain ranges and valleys, and leading VOCs of ecotypes

Ecotype number	Mountain range	Valley/sample short cut	Leading VOCs in ecotypes (with numbers of VOCs from Fig. 2 in parentheses)
1	Skorušina mountains	Blatná Valley on the border with West Tatras/OfHab	α-Pinene (1160), β-myrcene (1456), 1-heptene (210), methylcyclohexane (292), heptane (231), isobutenylcarbinol (302)
	High Tatras	Javorová Valley/OfJav	
	Belianske Tatras	Monková Valley/OfMon	
2	West Tatras	Roháčska Valley/OfRoh	2-Propenal (164), methyl propanol (Zázvorka 1997), isobutyronitrile (Schneeweiss 2007), 1-heptanol (738), 2,2-dimethylbutane (1235)
	Low Tatras—west	Svidovo Valley/OfSvi	
	Small Fatra—north	Bránica Valley/OfBra	
3	Big Fatra	Blatnická Valley/OfBla	Cumene (1103), isovaleraldehyde (115), benzene (132), 3-methylhexane (168), isopentyl acetate (717), pentanal (223)
	Oravské Beskydy	Polhoranka Valley/OfPol	
4	Low Tatras—east	Svarín Valley/OfSva	1-Undecene (2123), limonene (1826), eucalyptol (1880), dihydromyrcenol (2057), terpenyl acetate (3355), isopropyl palminate (5053)
5	Oravské Beskydy	Salt Water/OfSw	2-Butanamine (Gross et al. 2016), decanoic acid (3336), 2-nonenal (2532), dibuthyl phthalate (5021), nonanoic acid (2981), phenoxyethanol (2902)
6	Small Fatra—south	Lesná Valley/OfLes	Methylcyclohexyl acetate (250), 1-methoxypropan-2-ol (157), butanol (127), cyclohexanone (1142), acetone (Borges et al. 2013), (*E*)-2-methyl-2-butenal (143)

Hierarchical clustering analysis confirmed the separate clustering of ecotypes as shown in the PCA (Fig. 3.). The approximately unbiased *P*-value (AU, lower red numbers) obtained using Pvclust (a better approximation of clustering reliability than the bootstrap probability *P*-value, BP, upper green numbers) shows that most clusters within the dataset are reliable and significant.

**Figure 4:**
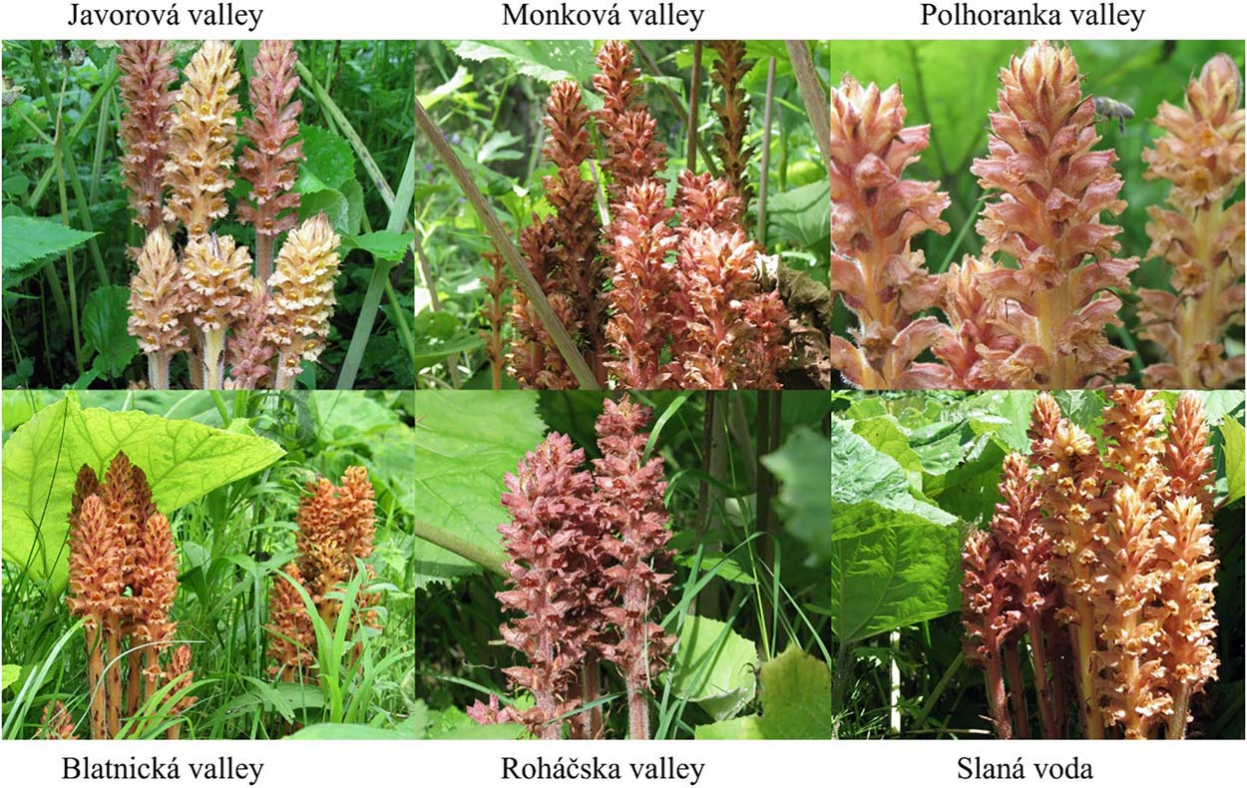
Colored morphs of *O. flava* in different mountain valleys of Slovakia (photo P. Tóth, 2010–2012).

Based on these data, we conclude that the *O. flava* plants growing at the various sites investigated belonged to different ecotypes (Fig. 2.). In addition to the VOC profiles, the phenotypes of the plants also differ between sites (Fig. 4), or even within one site (Supplementary Fig. S1). The flower color phenotype is the chemically characterized part of the population, where morphologically indistinguishable individuals of *O. flava* are associated with the same color. Chemotype refers to a group of morphologically identical individuals within a population that can be distinguished by their chemical composition, specifically regarding the chemical characteristics of their flower color. This means that while two plants may look the same (morphology), they may produce different pigments or different ratios of pigments, resulting in different flower colors, and thus different chemotypes (Abdala-Roberts and Moreira 2024).

At lower altitudes and in habitats with more diverse flora and sun, there were more pollinators, both in terms of species and numbers. The few examples of honeybees were recorded, for example, in Blatnická Valley, Lesná Valley, Svarín Valley, Polhoranka Valley, and Salt water (Supplementary Fig. S2). The most important pollinators of *O. flava* in Slovakia are listed in Table 2.

**Table 2:** List of pollinators across the sites with occurrence of *O. flava* in Slovakia during 2009–2012

Species/family	Name of species
Bumblebees/Apidae (Supplementary Fig. S4)	
	*Bombus hypnorum*
	*Bombus lapidarius*
	*Bombus lucorum*
	*Bombus pascuorum*
	*Bombus pratorum*
	*Bombus pyrenaeus*
	*Bombus terrestris*
	*Bombus wurflenii*
Honeybees/Apidae (Supplementary Fig. S4)	
	*Apis mellifera*
Wasps/Vespidae (Supplementary Fig. S5)	
	*Dolichovespula norwegica*
	*Dolichovespula sylvestris*
	*Vespula rufa*
	*Symmorphus gracilis*
Solitary bees/sweet bees (Halictidae) and polyester bees (Colletidae) (Supplementary Fig. S6)	
	*Halictus tumulorum*
	*Hylaeus confusus*
	*Hylaeus difformis*
Hoverflies/Syrphidae (Supplementary Fig. S7)	
	*Bacca elongata*
	*Episyrphus balteatus*
	*Melanostoma mellinum*
	*Melanostoma scalare*
	*Platycheirus albimanus*
	*Sphegina clunipes*

## Discussion

We studied the interactions of holoparasitic broomrapes in relation to their natural environment to increase our current understanding of floral scent and its roles in plant life. In this study 135 VOCs were identified in the emitted scents of the butterbur broomrape (*O. flava*), including a core set of toluene, methyl isovalerate, methyl ester, ethylbenzene, styrene, and isopropyl myristate. Plant volatiles are a key phenotype that mediates a myriad of ecological interactions in natural and managed systems, including pollination (Rering et al. 2018). Although floral scent is considered a crucial pollinator attractant, it remains relatively poorly understood compared to other components of the floral phenotype, such as flower color and morphology. Indeed, floral fragrance is not easily quantified and therefore data in the literature are scarce. This may be attributed to the nature of smell itself, which is less obvious than visual cues and requires specialized equipment and interdisciplinary skills to investigate. However, during the last two decades, the development of highly efficient tools in plant chemical analyses and insect sensory biology has led to a growing body of literature on floral scents and their variation (Fenster et al. 2004, Dicke and Takken 2006, R.A. Raguso 2008a, 2008b, Whitehead and Peakall 2009). Our results show a clear difference in the floral VOC phenotypes of *O. flava* plants that grew in different locations. In lowland and mountain regions, tens of kilometers apart, they emitted different proportions of volatile substances. Based on the various proportions of VOCs, it can be concluded that these are different ecotypes of *O. flava*. The term “ecotype” was proposed by Turesson in 1922 (G. Turesson 1922a, 1922b) and subsequently used to indicate groups of populations (ecological races, subspecies) in relation to the type of habitat or climate. The term is now used by ecologists to refer to almost any degree of genetic variation below the species level (Quinn 1978). A remarkable finding is that in the lower mountain valleys, for example in the Blatnická and Lesná valleys, the broomrapes were pollinated by several types of pollinators: bumblebees, wasps, and solitary bees. In general, the most widely used explanation for variation in floral characters and especially floral scents is selection mediated by pollinators (i.e. an evolutionary process that occurs in flowering plants in which the foraging behavior of pollinators is differentially selected for certain floral traits, such as floral rewards, but also flower size, shape, color, and scent) (Fenster et al. 2004). Pollinator-mediated selection can lead to intraspecific variation in floral characters on geographic and/or temporal scales. Variation in floral volatiles has been shown to coincide with variation in pollinator identities (Chess et al. 2008, Schlumpberger and Raguso 2008, Breitkopf et al. 2013, Doubleday et al. 2013, Gross et al. 2016). For example, two different ecotypes of the orchid *Ophrys sphegodes* that attract different pollinators displayed distinct VOC profiles, suggesting adaptation to locally available pollinators (Breitkopf et al. 2013). Or, in the case of the plant species *Gymnadenia odoratissima*, variations in floral scent between lowland and mountain populations corresponded to changes in pollinator assemblages (Gross et al. 2016). VOC emission rates are known to vary throughout the life of plant flowers. For example, when the fragrance compositions of different flower stages were analyzed, significant differences in the proportion of VOCs were found (Schiestl and Ayasse 2001, Borges et al. 2013). In some cases, an increase in the proportion of some compounds was observed after pollination, which could act as a repellent, either preventing further pollinator visits or deterring enemies, and/or allowing pollinators to be directed to unpollinated flowers (Raguso et al. 2003, Proffit et al. 2008). The results of our study indicate that the divergence (i.e. the increasing divergence of the characters of mutually isolated groups of organisms of the same origin during phylogenetic evolution) of volatile compounds in *O. flava* may be the result of adaptation to which they vary by altitude, biotype, and available pollinators. In plants, one or a few key compounds in the complex VOC blend have often been shown to contain all the information required for a given interaction. The scent of plants is usually composed of volatile substances, each of which has distinct functions independent of the ratio to other compounds, forming a multifunctional scent (Junker et al. 2018). Since pollinator attraction is typically only mediated by a fraction of the VOCs present in the scent, some of the observed variation may be pollinator-neutral (Raguso et al. 2003, Huber et al. 2005, Dötterl et al. 2007, Salzmann et al. 2007, Svensson et al. 2010). For example, in *Silene* (*Silene latifolia*), out of the floral VOCs that show remarkable differences between populations in Europe and North America, only one compound (lilac aldehyde) appears to be responsible for most of the attraction of the main pollinator (the *Hadena* moth) in European populations (Dötterl et al. 2005, 2006). This could indicate that all the other VOCs are neutral in terms of pollinator attraction. In some deceptive pollination systems, floral VOCs have been found to mimic pollinator pheromones, and any geographic difference in pheromone composition could lead to variation in floral scents through adaptation to local pollinator species (Mant et al. 2005). This could potentially explain the adaptations to the floral scent of the butterbur broomrapes in mountain areas to contain higher levels of α- and β-pinene and β-myrcene. At higher altitudes, the usual pollinators of *O. flava* are no longer available, while the available hoverflies have been reported to be attracted by these three VOCs (Stökl et al. 2011). On the other hand, bumblebees perceive many volatile compounds (Dötterl and Schäffler 2007), especially monoterpenes (Dekeboet al. 2022, Slavković and Bendahmane 2023), for example limonene, eucalyptol, terpenyl acetate, *trans*-linalool 1-oxide and we found also alcohols occurring on lower locations as butanol or 1-methoxypropan-2-ol regardless of location. This all supports a significant role for pollinator-mediated selection in the evolution of floral VOCs (Steiner et al. 2011, Stökl et al. 2011). In addition to pollinator-mediated selection, there are other factors that explain the variation in floral scents in the VOC blend of different ecotypes of a single species in different locations. Some studies have demonstrated changes in the VOC phenotype in relation to various abiotic factors, such as moisture, temperature, and light. However, changes in VOC emission do not necessarily indicate a lack of adaptation. This suggests the plasticity of VOC emissions in response to environmental factors (Knudsen 1994, Dötterl et al. 2005, Majetic et al. 2009, Falara et al. 2013, Farré-Armengol et al. 2014, 2015). Our findings also reveal slight variations in the color phenotypes of the broomrapes between and within locations. Changes in biochemical processes can explain floral VOC variation. This can potentially lead to a specific combination of color and scent associations. Several chemotypes (i.e. chemically characterized parts of a population of morphologically indistinguishable individuals) may be associated with the same color phenotype (Olesen and Knudsen 1994, Majetic et al. 2007, 2008, Keefover-Ring et al. 2009, Dormont et al. 2010, 2014, Delle-Vedove et al. 2011). As chemotypes are heritable, they can provide plants with a reliable source of information for kin recognition (R. Karban et al. 2014b).

In conclusion, butterbur broomrape *O. flava* ecotypes have adapted to a range of ecosystems and the corresponding local pollinator communities, with changes in their floral VOC blend. In this way, *O. flava* maintained its reproductive success, also in new ecological niches, by using local pollinators.

## Materials and Methods

### Plant material

**Figure 5:**
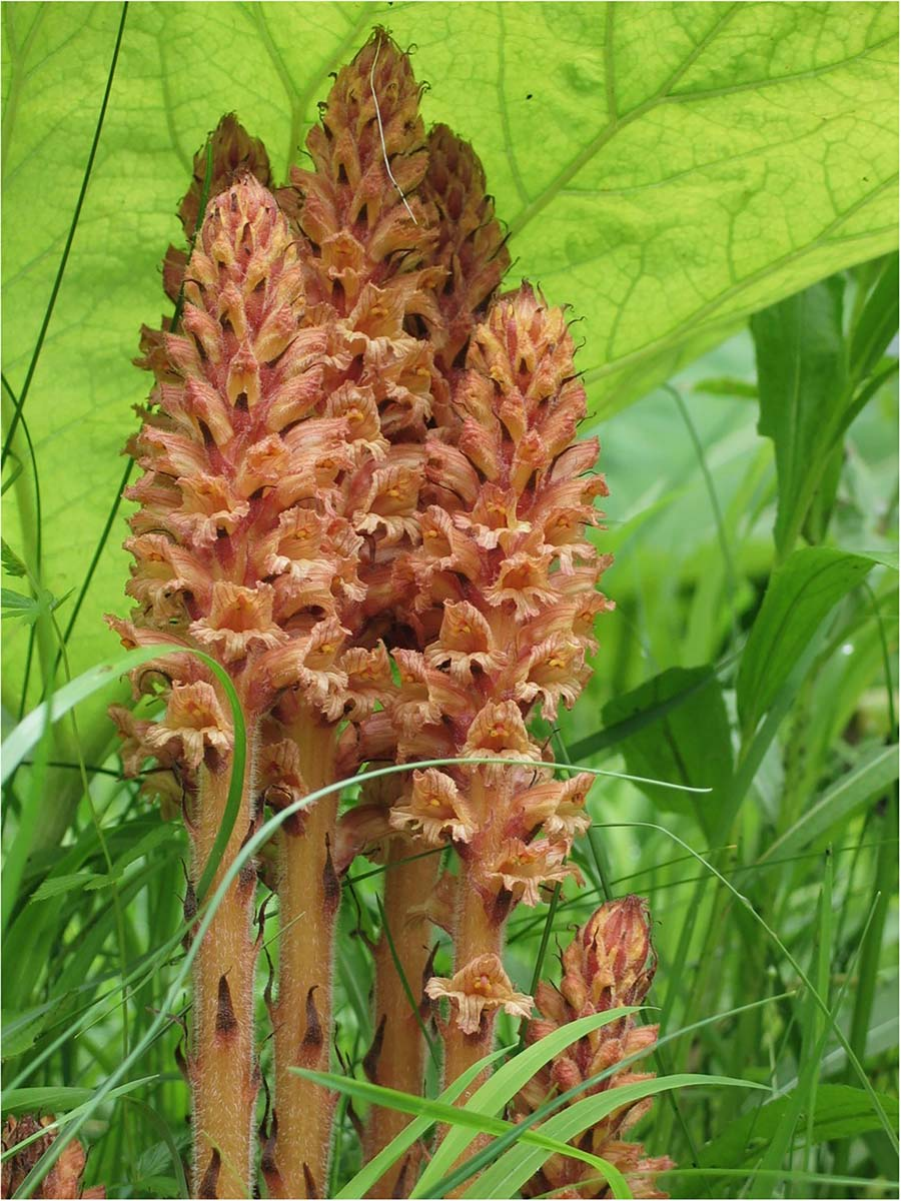
Butterbur broomrape—*O. flava* (Orobanchaceae), Blatnická Valley, Slovakia (photo P. Tóth 2010).

**Figure 6:**
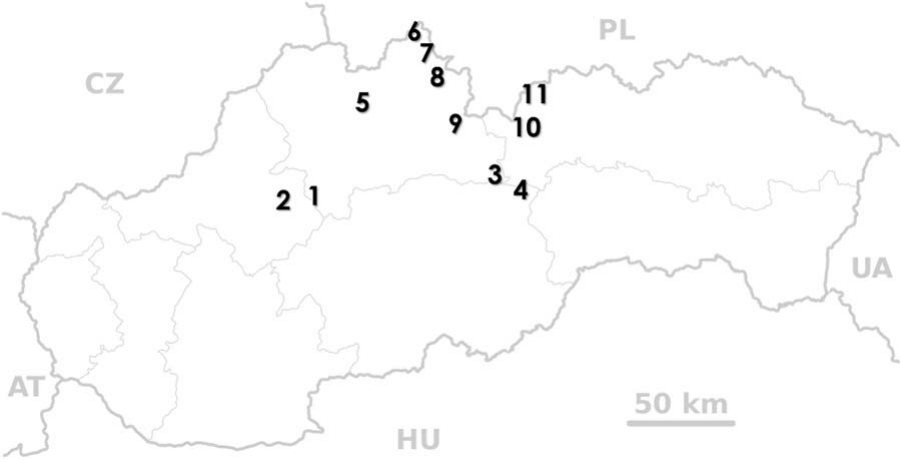
Location of study places in Slovakia during 2009–2012 (figure updated by P. Tóth). Notes: 1—Blatnická Valley; 2—Lesná valley; 3—Svidovo Valley; 4—Svarín Valley; 5—Bránica Valley; 6—Polhoranka Valley; 7—Salt water, –meadow; 8—Blatná Valley; 9—Roháčska Valley; 10—Monková Valley; 11—Javorová Valley.

Butterbur broomrape (*O. flava*) (Fig. 5) was identified in 11 separate locations in the Slovak Republic (Fig. 6). Specimens were identified according to the keys to species identification (e.g. Zázvorka 1997), and in consultation with expert Renata Piwowarczyk (personal communication) (Botany Department, Institute of Biology, Jan Kochanowski University, Kielce, Poland). Geographical details of the sites, including site names and broomrape host plants, are listed in Table 3.

**Table 3:** List of locations with occurrence of *O. flava* included in the study with host plant species and geographical origin in Slovakia during 2009–2012

Sample short cuts	Location name	GPS	Altitude (m)	Distance (km) ^l^	*Orobanche* host
OfBra1	^a^Bránica Valley	49°12′ N 18°59′ E	740	37	*Petasites mix*
OfBra2	^a^Bránica Valley	49°12′ N 18°59′ E	740	37	*P. mix*
OfBra3	^a^Bránica Valley	49°12′ N 18°59′ E	740	37	*P. mix*
OfBra4	^a^Bránica Valley	49°12′ N 18°59′ E	740	37	*P. mix*
OfLes1	^b^Lesná Valley	49°01′ N 18°38′ E	542	27	*Petasites hybridus*
OfLes2	^b^Lesná Valley	49°01′ N 18°38′ E	542	27	*P. hybridus*
OfLes3	^b^Lesná Valley	49°01′ N 18°38′ E	542	27	*P. hybridus*
OfBla1	^c^Blatnická Valley	48°54′ N 18°57′ E	667	0	*Petasites albus*
OfBla2	^c^Blatnická Valley	48°54′ N 18°57′ E	667	0	*P. albus*
OfBla3	^c^Blatnická Valley	48°54′ N 18°57′ E	667	0	*P. albus*
OfBla4	^c^Blatnická Valley	48°54′ N 18°57′ E	667	0	*P. albus*
OfSva1	^d^Svarín Valley	49°00′ N 19°52′ E	721	67	*P. mix*
OfSva2	^d^Svarín Valley	49°00′ N 19°52′ E	721	67	*P. mix*
OfSva3	^d^Svarín Valley	49°00′ N 19°52′ E	721	67	*P. mix*
OfSva4	^d^Svarín Valley	49°00′ N 19°52′ E	721	67	*P. mix*
OfSvi1	^e^Svidovo Valley	48°58′ N 19°43′ E	938	59	*P. albus*
OfSvi2	^e^Svidovo Valley	48°58′ N 19°43′ E	938	59	*P. albus*
OfSvi3	^e^Svidovo Valley	48°58′ N 19°43′ E	938	59	*P. albus*
OfSvi4	^e^Svidovo Valley	48°58′ N 19°43′ E	938	59	*P. albus*
OfHab1	^f^Blatná Valley	49°17′ N 19°42′ E	940	68	*P. albus*
OfHab2	^f^Blatná Valley	49°17′ N 19°42′ E	940	68	*P. albus*
OfHab3	^f^Blatná Valley	49°17′ N 19°42′ E	940	68	*P. albus*
OfHab4	^f^Blatná Valley	49°17′ N 19°42′ E	940	68	*P. albus*
OfJav1	^g^Javorová Valley	49°14′ N 20°08′ E	911	96	*Tussilago farfara*
OfJav2	^g^Javorová Valley	49°14′ N 20°08′ E	911	96	*T. farfara*
OfJav3	^g^Javorová Valley	49°14′ N 20°08′ E	911	96	*T. farfara*
OfJav4	^g^Javorová Valley	49°14′ N 20°08′ E	911	96	*T. farfara*
OfMon1	^h^Monková Valley	49°16′ N 20°15′ E	914	103	*P. albus*
OfMon2	^h^Monková Valley	49°15′ N 20°15′ E	914	103	*P. albus*
OfMon3	^h^Monková Valley	49°16′ N 20°15′ E	914	103	*P. albus*
OfMon4	^h^Monková Valley	49°15′ N 20°15′ E	914	103	*P. albus*
OfPol1	^i^Polhoranka Valley	49°33′ N 19°24′ E	725	79	*P. albus*
OfPol2	^i^Polhoranka Valley	49°33′ N 19°24′ E	725	79	*P. albus*
OfPol3	^i^Polhoranka Valley	49°33′ N 19°24′ E	725	79	*P. albus*
OfPol4	^i^Polhoranka Valley	49°33′ N 19°24′ E	725	79	*P. albus*
OfSw1	^j^Salt water	49°31′ N 19°28′ E	752	77	*P. albus*
OfSw2	^j^Salt water	49°31′ N 19°28′ E	752	77	*P. albus*
OfSw3	^j^Salt water	49°31′ N 19°28′ E	752	77	*P. albus*
OfSw4	^j^Salt water	49°31′ N 19°28′ E	752	77	*P. albus*
OfRoh1	^k^Roháčska Valley	49°15′ N 19°42′ E	1001	66	*P. albus*
OfRoh2	^k^Roháčska Valley	49°15′ N 19°42′ E	1001	66	*P. albus*
OfRoh3	^k^Roháčska Valley	49°15′ N 19°42′ E	1001	66	*P. albus*
OfRoh4	^k^Roháčska Valley	49°15′ N 19°42′ E	1001	66	*P. albus*

a Mountain range—Small Fatra, north.

b Mountain range—Small Fatra, south.

c Mountain range—Big Fatra.

d Mountain range—Low Tatras, east.

e Mountain range—Low Tatras, west.

f Mountain range—Skorušina Mountain—valley between West Tatras and Skorušina mountains.

g Mountain range—High Tatras.

h Mountain range—Belianske Tatras.

i Mountain range—Oravské Beskydy—valley.

j Mountain range—Oravské Beskydy—meadow.

k Mountain range—West Tatras—valley.

l Air distance from Blatnická Valley.

### Headspace trapping

The VOCs emitted by flowers were collected using dynamic headspace sampling, a method previously described by Tóth et al. (2016). Two or three flowering shoots per ecotype were excised, placed into vials filled with water, and immediately transferred to a glass jar (720 mL), which was tightly closed with a Teflon lid with an in- and outlet. The air was drawn from the jar using the portable PAS-500 Micro Air Sampler (Supelco, Sigma-Aldrich) with a low-flow orifice. The air flow was regulated by a flow controller (Brooks Instruments, Veenendaal, the Netherlands), which was set to ~100 mL/min. Incoming air was purified through stainless-steel cartridges (Markes, Llantrisant, UK) containing 200 mg Tenax TA (20/35 mesh; Grace-Ltech, Breda, the Netherlands). VOCs released by the flowers were trapped from the outgoing air on a similar cartridge for a period of 5 h, predominantly during between 10 a.m. and 3 p.m. Four collections and their respective controls (empty glass jars) were conducted simultaneously. After sampling, the Tenax cartridges were sealed and stored at room temperature until analysis.

### Gas chromatography

Subsequently, samples of trapped VOCs were analyzed at the Laboratory of Plant Physiology, Wageningen University, the Netherlands, using an ultrasensitive gas chromatography mass spectrometer (Trace Ultra GC) coupled to a Thermo Trace DSQ quadrupole mass spectrometer (Interscience, the Netherlands). This methodology was described in detail by Snoeren et al. (2010) and Tóth et al. (2016). The trapped VOCs were released from the Tenax by subjecting the cartridge to a heating process at 250°C for a period of 4 min, with a helium flow rate of 30 mL per minute. Following this, the compounds were retrapped on a multibed sorbent at 10°C. The heating of this sorbent enabled the compounds to be injected into a capillary column (RTX-5MS, 30 m, 0.25 μm id, 1.4 μm df, Restek, Interscience, the Netherlands) using the GC temperature programme, which commenced at 60°C (for a duration of 2.5 min) and subsequently increased to 250°C at a rate of 12°C/min (for a duration of 1.5 min) with a column flow of 1 mL/min. The column effluent was subjected to electron impact ionization at an energy level of 70 eV. Mass spectra were collected over the mass range *m/z* 35–300 at a scan rate of 3 spectra per second. The resulting chromatographic and spectral data were then evaluated using Xcalibur software (version 2.0.7, Thermo Fisher Scientific Inc.). The annotation of compounds was conducted through a comparative analysis of their mass spectra with those present in mass spectral libraries, specifically the Wiley 7th edition and the NIST08 database. Additionally, the calculated retention indices were compared with those provided in the NIST08, Adams (2007), and El-Sayed (2024) references. The annotation of numerous compounds was corroborated through the utilization of an in-house-developed mass spectra/retention index library. Any compounds exhibiting any atypical floral compounds were subjected to a rigorous examination of their documented biological properties and occurrence in other plants, as referenced in The Pherobase (El-Sayed 2024). This process was undertaken to ascertain their classification as flower VOCs or, at the very least, potential volatiles, given the dearth of data pertaining to their association with broomrapes in the existing literature.

### Insect observation

In addition to VOC sampling, the pollinators that visited *O. flava* at each site were analyzed during sunny weather, absence of rain and wind, and mild temperatures. Hereto, in 2009 11 ecotypes known for each of these sites were monitored for 5 h, from 10 a.m. to 3 p.m. This analysis was repeated in 2012. All the representative insects were recorded and were caught by sweeping or direct capture. Species identification (verification) was done with the help of pollinator expert Jozef Lukáš, Faculty of Natural Sciences, Comenius University Bratislava.

### Statistical analysis

Prior to statistical analysis, chromatograms were corrected using MetAlign software version 010110 (Lommen 2009), MetAlign Output Transformer (METOT; Plant Research International, Wageningen, the Netherlands), and mass spectra were reconstructed using MsClust (Tikunov et al. 2005). Samples that were affected too much by the presence of polysiloxanes or water or had very low signal intensity were excluded from the data set.

All the initial data (VOC profiles) were then analyzed using PCA, which was performed on compounds whose peak heights were significantly different between plants from individual sites (*P* < 0.05 as determined by ANOVA) using GeneMath XT (v2.12, Applied Maths NV, Belgium). Hierarchical clustering analysis (HCA) with 10 000 bootstrap replicates was performed in R (v2.10.1; R Development Core Team) following Shimodaira (2004).

## Supplementary Material

Supplementary_Material_Figs_S1-S7_pcag023

Supplementary_Material_Table_Pcag023

Supplementary_data_pcag023

## Data Availability

All the data are available, including peer review.
