# Supplementary material for: The pollination syndrome of parasitic plants depends on the environment
Source: Plant Cell Physiol. 2026 Feb 17;67(4):648–58. doi: 10.1093/pcp/pcag023 (PMC13192492; doi:10.1093/pcp/pcag023)
Supplement: Supplementary_Material_Figs_S1-S7_pcag023 [file supplementary_material_figs_s1-s7_pcag023.docx]

**SUPPLEMENTARY MATERIAL**

**Figures**

**
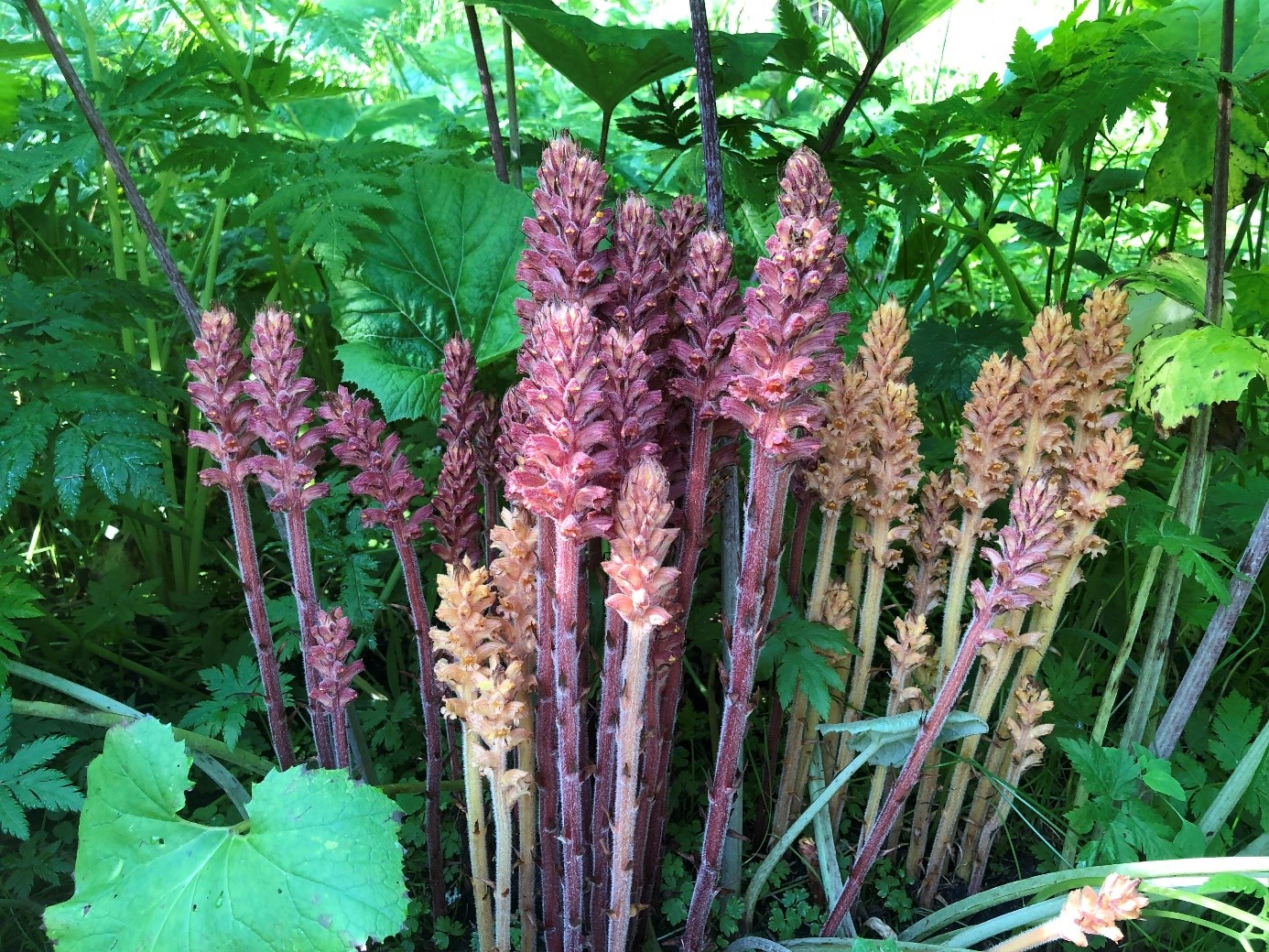
**

**Supplementary Figure 1.** Different color morphs of *Orobanche flava* within the same site, Roháčska valley, Slovakia (photo Peter Tóth 2010)


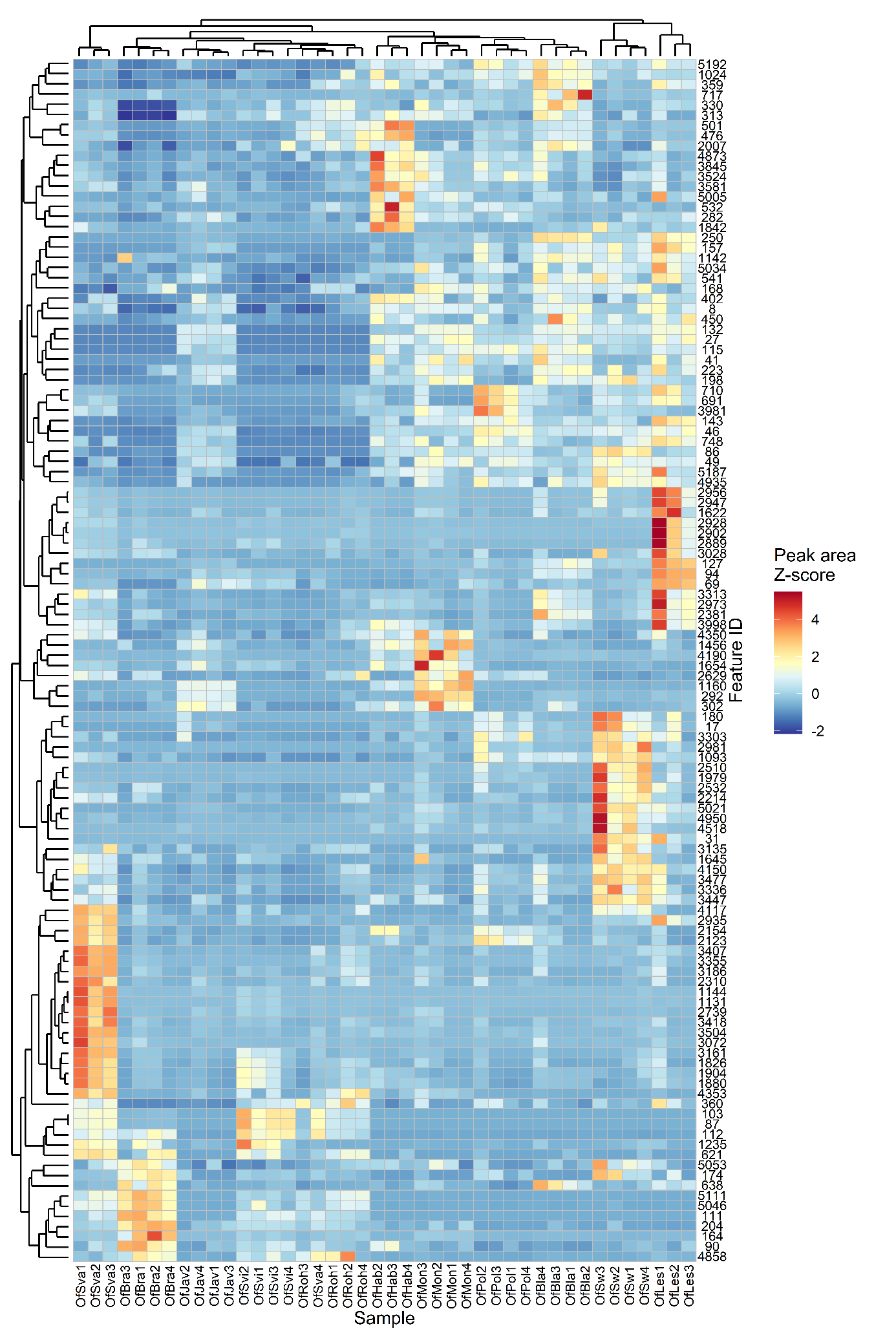


**Supplementary Figure 2.**

**Heatmap:** Normalized peak areas of GS-MS features detected in floral emissions of *Orobanche flava* flowers at different locations of Slovakia.

**Footnotes:**

**X axis**: shortcuts of Slovak study sites, from left, OfSva – Svarín valley, OfBra – Bránica valley, OfJav – Javorová valley, OfSvi – Svidovo valley, OfRoh – Roháčska valley, OfHab – Blatná valley, OfMon – Monková valley, OfPol – Polhoranka valley, OfBla – Blatnická valley, OfSw – Salt Water, OfLes – Lesná valley

**Y axis**: numbers represented the numbers of key volatile organic compounds for single ecotypes.

**Supplementary material,** Excel file (see separate excel file) which represents **PCA matrix:** Scores, loadings, and explained variances of PCA analysis of *Orobanche flava* floral volatile profiles; shortcuts are the same as in the figure with heatmap


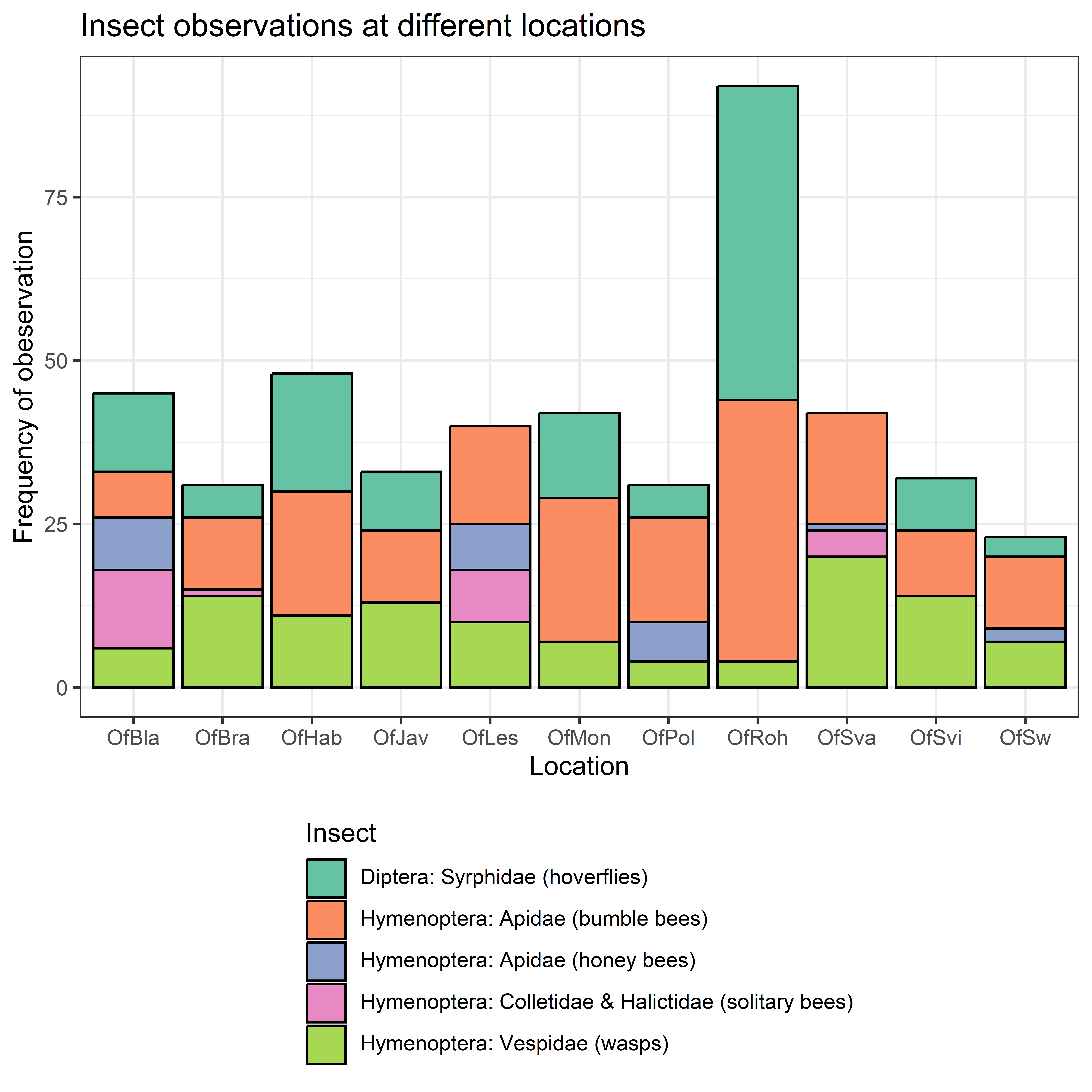


**Supplementary Figure 3.** Structure of the pollinator guild on *Orobanche flava* in Slovak mountain localities of different altitudes, showing summed insect numbers during 2009 - 2012.

OfBla – Blatnická valley (667 m a.s.l.), OfBra – Bránica valley (740 m a.s.l.), OfHab – Blatná valley (940 m a.s.l), OfJav – Javorová valley (911 m a.s.l.), OfLes – Lesná valley (542 m a.s.l.), OfMon – Monková valley (914 m a.s.sl.), OfPol – Polhoranka valley (725 m a.s.l.), OfRoh – Roháčska valley (1001 m a.s.l.), OfSva – Svarín valley (721 m a.s.l.), OfSvi – Svidovo valley (938 m a.s.l.), OfSw – Slaná voda (752 m a.s.l.)


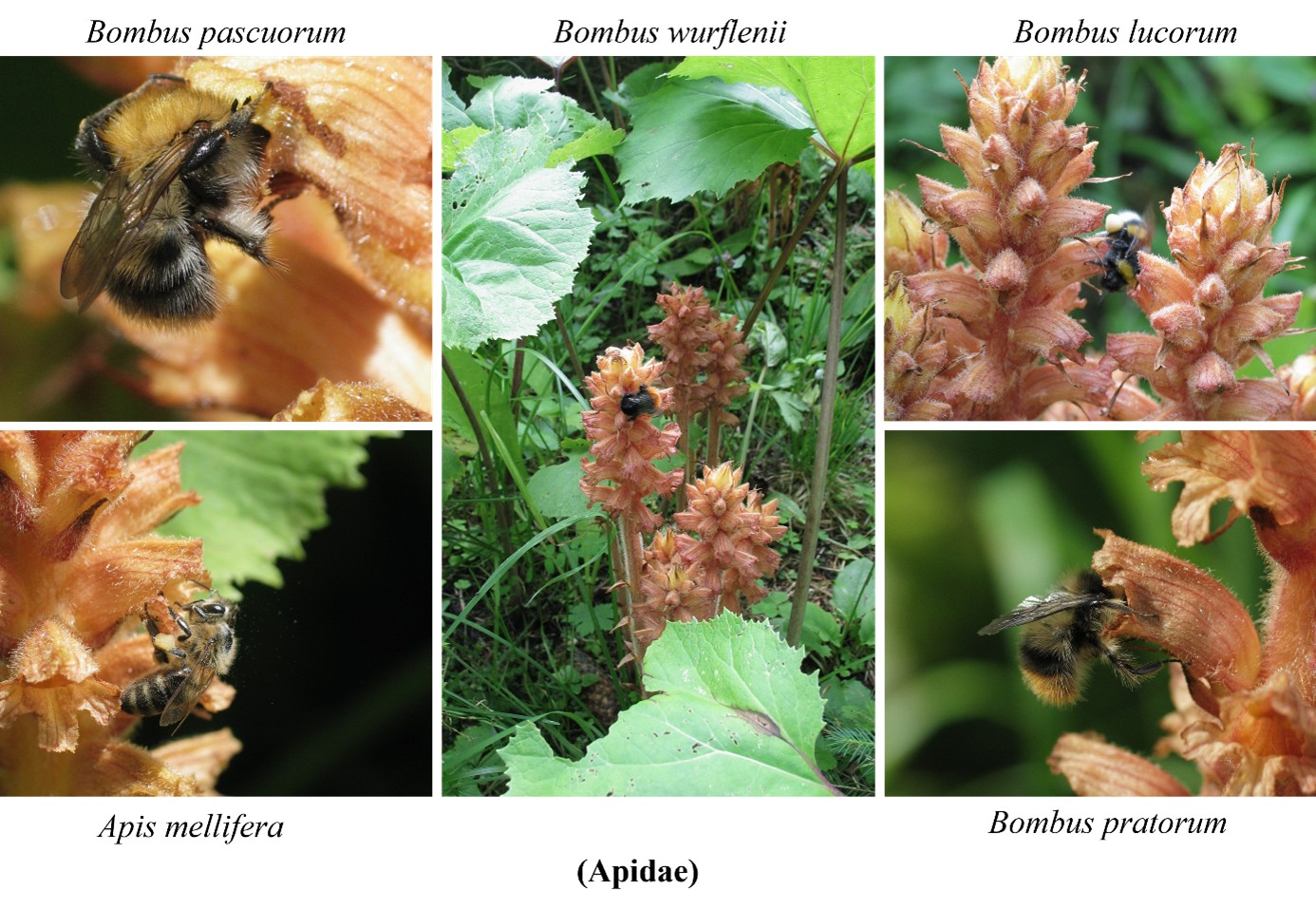


**Supplementary Figure 4.** Bumblebees, *Bombus* spp. (Hymenoptera: Apidae) on flowers of *Orobanche flava* at Roháčska valley and European honeybee, *Apis mellifera* (Hymenoptera: Apidae) on flowers of *O. flava* at Polhoranka valley, Slovakia (photo Peter Tóth 2009-2012)


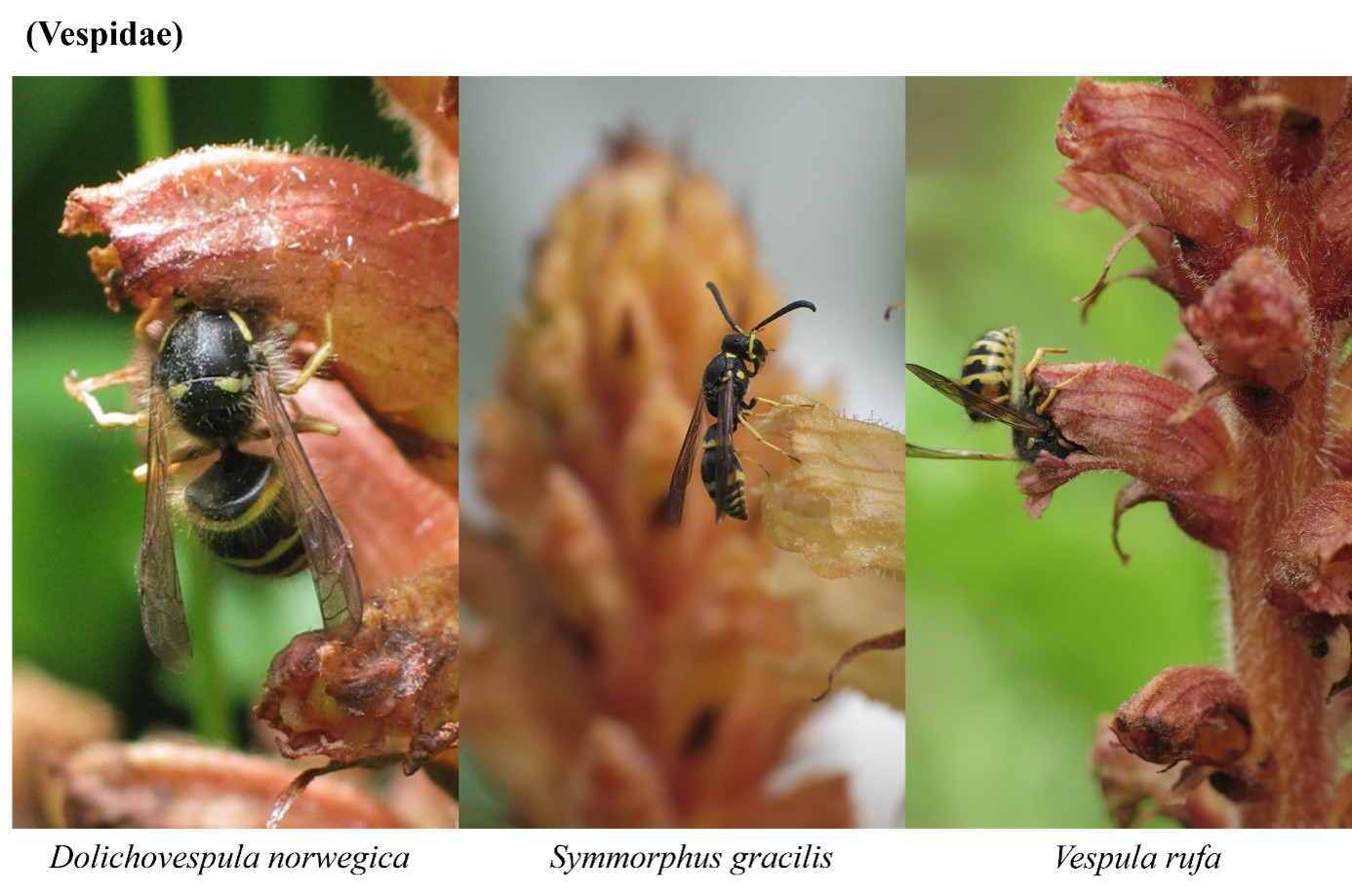


**Supplementary Figure 5.** Wasps (Hymenoptera: Vespidae) on *Orobanche flava* at Roháčska valley only *Symmorphus gracilis* at Blatnická valley, Slovakia (photo Peter Tóth 2009-2012)


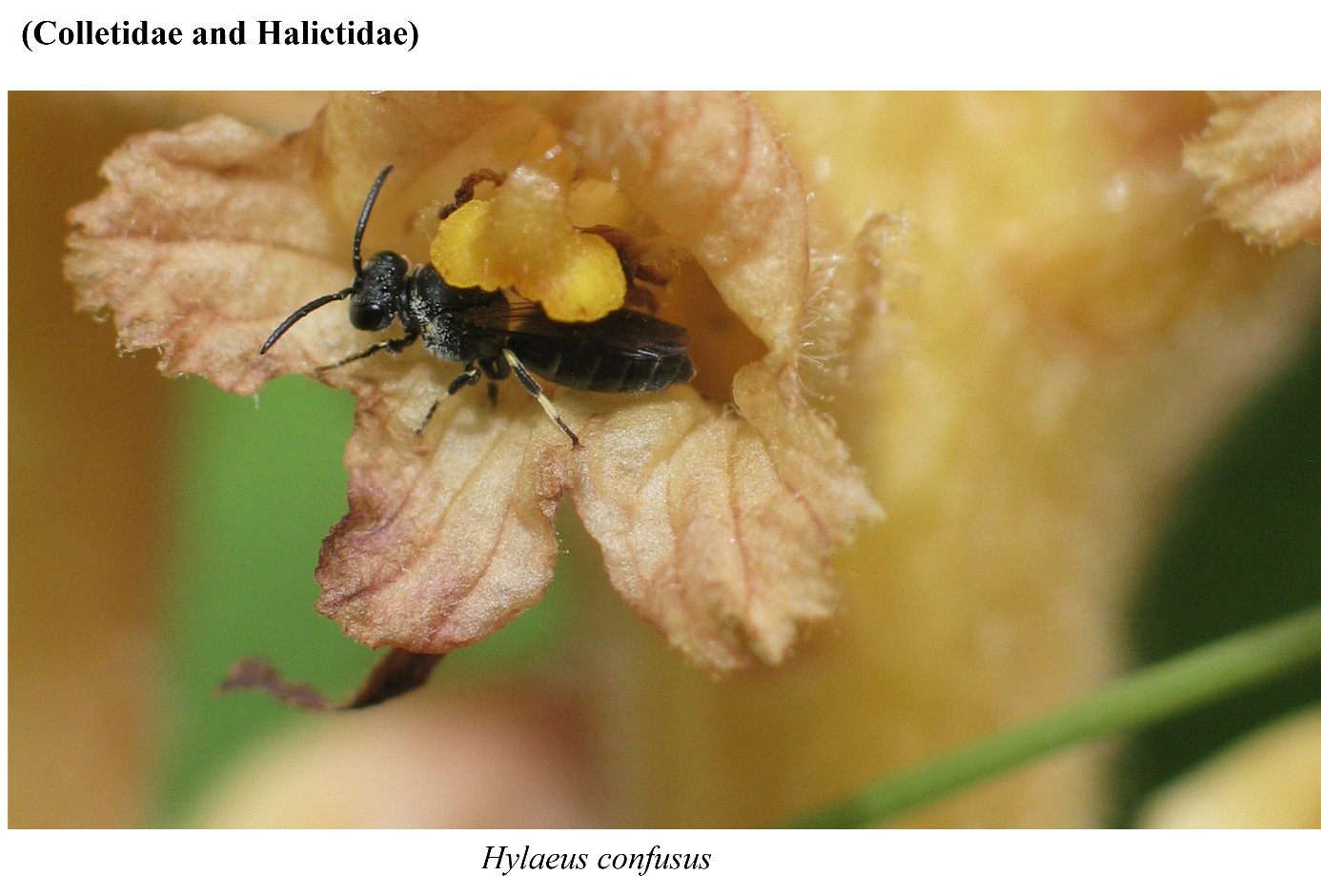


**Supplementary Figure 6.** Sweet bee *Hylaeus confusus* (Hymenoptera: Colletidae) with tendency to be solitary; here on flower of *Orobanche flava* at Blatnická valley, Slovakia **(**photo Peter Tóth 2012)


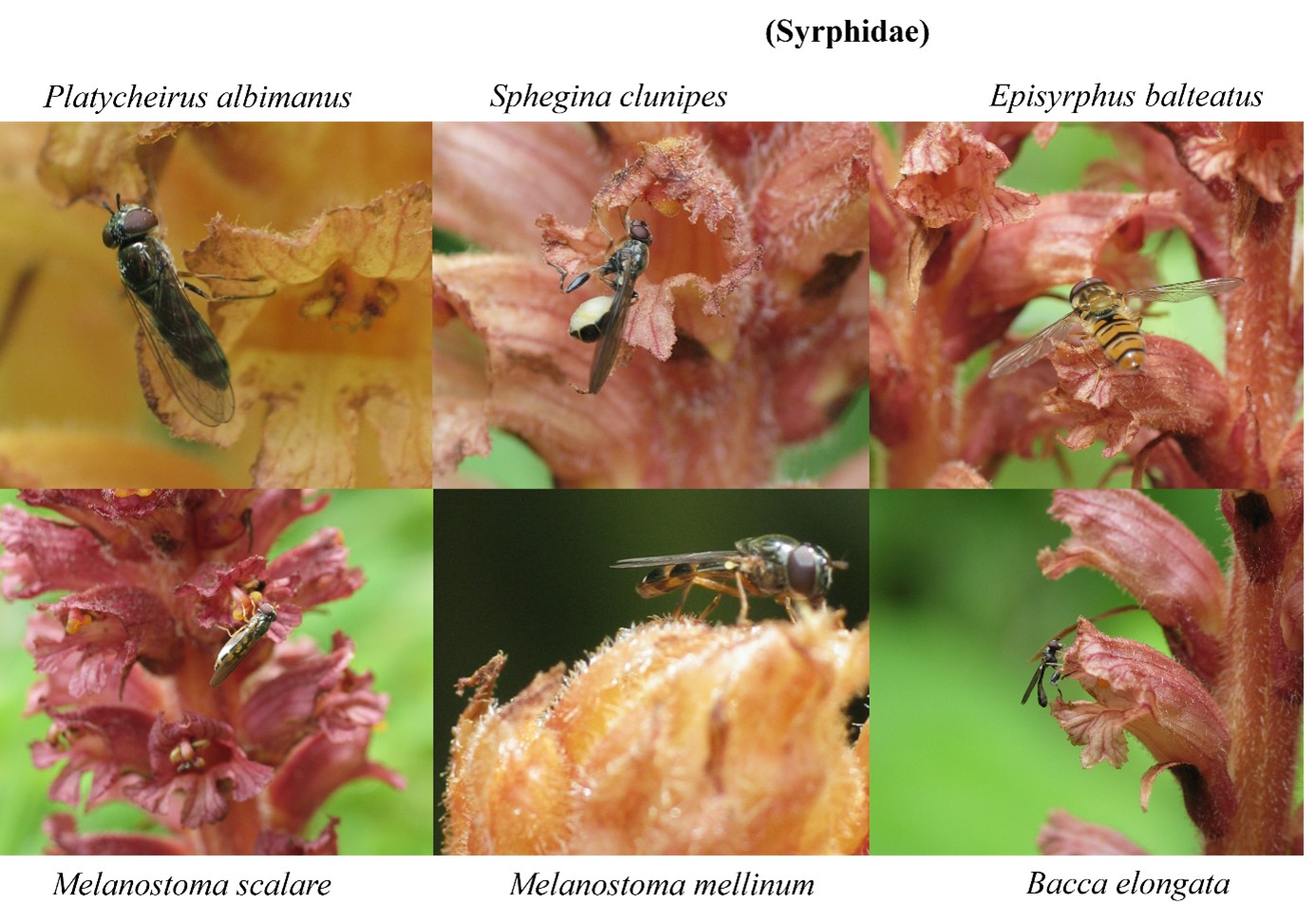


**Supplementary Figure 7.** Hoverflies (Diptera: Syrphidae) on flowers of *Orobanche flava* at Monková and Roháčska valleys, Slovakia; the most common at mountains valleys was *Platycheirus albimanus* (photo Peter Tóth 2009-2012)
