# Supplementary material for: The pollination syndrome of parasitic plants depends on the environment
Source: Plant Cell Physiol. 2026 Feb 17;67(4):648–58. doi: 10.1093/pcp/pcag023 (PMC13192492; doi:10.1093/pcp/pcag023)
Supplement: Supplementary_Material_Table_Pcag023 [file supplementary_material_table_pcag023.docx]

**Supplementary material_PTóth_et_al._December_2025**

**Tables**

**Supplementary Table 1.** List of floral volatile organic compounds emitted by *Orobanche flava* ordered according to calculated retention indexes (RI).

| RI^a^ | Volatile organic compound^, c^ |
| --- | --- |
| 461 | Ethyl alcohol* |
| 487 | Propan-2-one* |
| 583 | Acetic acid * |
| 672 | Ethyl acetate* |
| 675 | 2-Methylpropan-1-ol (isobutanol)* |
| 686 | 3-Methylbutanal * |
| 688 | Butan-1-ol * |
| 689 | 2-Methylbutanal |
| 690 | 2-Methyl-2-butenal |
| 691 | 2,3-Dihydro-5-methylfuran |
| 692 | 1-Methoxypropan-2-ol* |
| 694 | 1-Penten-3-ol* |
| 696 | Pentan-2-one |
| 698 | 1-Hepten |
| 699 | Pentanal* |
| 700 | Heptane* |
| 701 | 2-methyl-1,4-hexadiene |
| 708 | 3-Hydroxybutan-2-one (acetoin)* |
| 718 | 2,4-Dimethylfuran* |
| 725 | 2-Methylbutanenitrile |
| 731 | 1,1-Diethoxyethane |
| 734 | 3-Methylbutan-1-ol* |
| 739 | 2-Methylbutan-1-ol* |
| 742 | 4-Methylpentan-2-ol* |
| 746 | 2-Methyl-2-butenal* |
| 767 | Pentan-1-ol* |
| 774 | Toluene* |
| 778 | Methyl 3-methylbutanoate* |
| 784 | Pentane-2,4-dione* |
| 787 | 3-Methyl-2-butenal* |
| 791 | Hexan-2-one |
| 793 | 1-Octene* |
| 798 | Hexanal* |
| 813 | Butyl acetate |
| 824 | 2,4-Dimethylheptane* |
| 833 | 3-Methylbutanoic acid |
| 845 | 2,4-Dimethylheptene |
| 849 | 3-Methylbutyl acetate |
| 854 | 2-Methylbutanal oxime |
| 860 | cis-2,3-Epoxyoctane |
| 866 | 4-Methyloctane |
| 867 | Hexan-1-ol* |
| 870 | 1-Ethylbenzene* |
| 877 | 1,4-Dimethylbenzene* |
| 888 | Heptan-3-one* |
| 891 | Heptan-2-one |
| 895 | 1-Nonyne |
| 900 | Ethenylbenzene* |
| 903 | Heptanal* |
| 907 | 2-Butoxyethanol |
| 929 | 2,5-Hexanedione |
| 935 | 2,6-Dimethyloctane |
| 943 | 1-Butoxy-2-propanol |
| 947 | α-Pinene* |
| 954 | Isobutyl butyrate |
| 957 | 4-Ethyloctane |
| 964 | 4-Methylnonane |
| 969 | Heptan-1-ol |
| 972 | m-Ethylmethylbenzene |
| 973 | Benzaldehyde* |
| 978 | Pseudocumene |
| 979 | 1-Octen-3-ol* |
| 987 | 6-Methyl-5-hepten-2-one* |
| 992 | β-Myrcene* |
| 1000 | Decane* |
| 1005 | Octanal* |
| 1021 | 3-Carene |
| 1029 | 2-Ethylhexan-1-ol* |
| 1037 | p-Cymene* |
| 1037 | 1,2,3-Trimethylbenzene* |
| 1042 | Limonene* |
| 1043 | Methyl 2-ethylhexanoate |
| 1047 | Eucalyptol* |
| 1054 | 3-Methylbutyl butyrate |
| 1061 | 3,6-Dimethyldecane |
| 1067 | 2-Methyldecane |
| 1075 | Dihydromyrcenol |
| 1080 | 1-Phenylethanone* |
| 1100 | trans-Linalool oxide |
| 1103 | β-Linalool |
| 1108 | Nonanal* |
| 1145 | cis-Limonene oxide |
| 1171 | R-camphor* |
| 1175 | 2-Decen-1-ol* |
| 1178 | Sabina ketone |
| 1190 | Menthol |
| 1200 | Dodecane* |
| 1208 | Decanal* |
| 1214 | Naphthalene |
| 1223 | Itaconic acid diethylester* |
| 1232 | 4-tert-Butylcyclohexanol |
| 1238 | Fenchyl acetate |
| 1255 | Geraniol* |
| 1262 | Carvone |
| 1271 | 2-Butyloctan-1-ol |
| 1295 | Isopulegol acetate |
| 1300 | Tridecane* |
| 1307 | Limonene dioxide |
| 1311 | Isobornyl acetate |
| 1368 | Terpinyl acetate |
| 1372 | 2,6,8-Trimethyldecane |
| 1380 | Farnesan |
| 1400 | Tetradecane* |
| 1416 | Dodecanal* |
| 1422 | 2-Methyl-1-dodecanol |
| 1435 | 1-Phenoxybenzene |
| 1441 | 2-Methyl-Z-4-tetradecene |
| 1453 | Longifolene |
| 1458 | trans-Geranyl acetone* |
| 1469 | β-Cedrene |
| 1479 | Aristolene* |
| 1479 | Dodecan-1-ol |
| 1490 | 1-Pentadecene |
| 1500 | Pentadecane***** |
| 1509 | α-Cetone |
| 1516 | γ-Cadinene |
| 1554 | Lilial |
| 1592 | 2-Hexyl-1-octanol |
| 1600 | Hexadecane* |
| 1615 | α-Cedrene epoxide |
| 1627 | Isopropyl dodecanoate |
| 1681 | Clavatol |
| 1700 | Heptadecane* |
| 1789 | α-Hexylcinnamaldehyde |
| 1800 | Octadecane* |
| 1813 | Phytane |
| 1822 | Isopropyl tetradecanoate* |
| 1900 | Dibutyl phthalate* |
| 2000 | Eicosane* |
| 2029 | Isopropyl hexadecanoate* |
| 2100 | Heneicosane* |
| 2200 | Docosane* |
| 2300 | Tricosane* |
| 2400 | Tetracosane* |

**Notes**

^a^ VOCs are listed according to calculated Kovats retention indices (RI). Calculation was done for capillary column RTX-5MS based on retention times of alkanes

^b^ Asterisk* indicate VOCs which were checked by authentic standards and/or agree with mass spectral libraries

^c^ most of the compounds were annotated by comparing the mass spectra with mass spectral libraries (Wiley 7^th^ edition and NIST08), and by comparing calculated retention indices with those given by NIST08, Adams (2007) and El-Sayed (2024). The annotation of many compounds was verified using an in-house developed mass spectra/RI library at Wageningen UR.
